# A CT-based radiomics nomogram for distinguishing between benign and malignant bone tumours

**DOI:** 10.1186/s40644-021-00387-6

**Published:** 2021-02-06

**Authors:** Weikai Sun, Shunli Liu, Jia Guo, Song Liu, Dapeng Hao, Feng Hou, Hexiang Wang, Wenjian Xu

**Affiliations:** 1 Department of Radiology, The Affiliated Hospital of Qingdao University Qingdao, 16 Jiangsu Road, Qingdao, Shandong China; 2 Department of Pathology, The Affiliated Hospital of Qingdao University, Qingdao, Shandong China

**Keywords:** Computed tomography, Morphological feature, Nomogram, Differential diagnosis, Radiomics

## Abstract

**Background:**

We sought to evaluate the performance of a computed tomography (CT)-based radiomics nomogram we devised in distinguishing benign from malignant bone tumours.

**Methods:**

Two hundred and six patients with bone tumours were spilt into two groups: a training set (*n* = 155) and a validation set (*n* = 51). A feature extraction process based on 3D Slicer software was used to extract the radiomics features from unenhanced CT images, and least absolute shrinkage and selection operator logistic regression was used to calculate the radiomic score to generate a radiomics signature. A clinical model comprised demographics and CT features. A radiomics nomogram combined with the clinical model and the radiomics signature was constructed. The performance of the three models was comprehensively evaluated from three aspects: identification ability, accuracy, and clinical value, allowing for generation of an optimal prediction model.

**Results:**

The radiomics nomogram comprised clinical and radiomics signature features. The nomogram model displayed good performance in training and validation sets with areas under the curve of 0.917 and 0.823, respectively. The areas under the curve, decision curve analysis, and net reclassification improvement showed that the radiomics nomogram model could obtain better diagnostic performance than the clinical model and achieve greater clinical net benefits than the clinical and radiomics signature models alone.

**Conclusions:**

We constructed a combined nomogram comprising a clinical model and radiomics signature as a noninvasive preoperative prediction method to distinguish between benign and malignant bone tumours and assist treatment planning.

## Background

Distinguishing between benign and malignant bone tumours is crucial for clinical decision and treatment [1, 2]. Routine imaging examinations include radiography, computed tomography (CT), magnetic resonance imaging (MRI), bone scintigraphy, and positron-emission tomography/computed tomography [3]. Radiography is recommended as the first choice for the initial differential diagnosis of benign and malignant primary bone tumours; in particular, evaluation of the lesion edge is more accurate and effective than can be achieved using CT or MRI [4]. However, CT is helpful in the diagnosis of tumours that are easily affected by other anatomical sites and plays an important role in the formulation of a surgical plan. The reported accuracy of malignancy assessment by CT, assuming that equivocal findings are benign, is approximately 83% [5]. However, the radiographic appearance is often nonspecific and nondiagnostic.

Radiomics is an emerging method of medical image analysis. Its essence is to extract quantitative features from medical images and to use them to describe the characteristics of tissues and correlate the characteristics with patients’ prognosis [6]. Previous research has reported the feasibility of radiomics in discerning benign from malignancy tissues in small peripheral pulmonary nodules and breast lesions [7, 8]. However, research using radiomics nomograms for bone tumours is relatively limited. Textural analysis of CT imaging has been applied for assessment of bone lesions, but the accuracy was low (77.8–86%) [9–11]. These studies nonetheless provided a new approach to bone tumour diagnosis using quantitative imaging.

Our aim was to evaluate the performance of a radiomics nomogram derived from CT imaging in distinguishing between benign and malignant bone tumours.

## Methods

### Patients

Our institutional review board approved this retrospective study and the requirement for patient informed consent was waived. In total, 206 patients who underwent CT scans were pathologically diagnosed with bone tumours in our hospital from January 2008 to December 2018. The inclusion criteria were: (1) bone tumour confirmed by surgery and with complete pathological data. (2) the time interval between the CT examination and surgery was ≤2 weeks. The exclusion criteria were: (1) incomplete relevant clinical or pathological information; (2) insufficient CT or pathology quality with which to make a diagnosis. All cases were aggregated and divided into benign (*n* = 88) and malignant (*n* = 118) groups according to the pathology findings.

The diagnoses of the 206 lesions are presented in Table 1; 118 were malignant and 88 benign (117 males and 89 females; mean age 40.31 ± 21.28 y). Independent-samples *t*-test analysis showed that age significantly differed between the benign and malignant groups (*P* < 0.001). The 206 lesions were assigned to the training set (*n* = 155) and the validation set (*n* = 51) by stratified random sampling at a ratio of 3:1.

**Table 1:** Summary of 206 bone tumour confirmed by histologic results

Benign mass (*N* = 88)	Number	Malignant mass (*N* = 118)	Number
Aneurysmal bone cyst	3	Undifferentiated pleomorphic sarcoma	1
Non-ossifying fibroma	5	Giant cell tumor	20
Ossifying fibroma	11	Osteosarcoma	20
Osteoblastoma	2	Chordoma	16
Simple bone cyst	1	Myeloma	5
Osteochondroma	36	Langerhans cell histiocytosis	2
Osteofibrous dysplasia	11	Lymphoma	1
Osteoid osteoma	2	Chondrosarcoma	20
Enchondroma	5	Fibrosarcoma of bone	4
Chondroblastoma	8	Ewing sarcoma	3
Brown tumor	1	Bone metastasis	26
Hemangioma	1		
Intraosseous lipoma	1		
Myofibroblastoma	1		

### Image acquisition and segmentation of lesions

All CT scans were conducted on one of the following devices: BrightSpeed RT 16 Elite, LightSpeed CT750 HD (GE Healthcare, Milwaukee WI, USA) and SOMATOM Sensation 64 (Siemens, Forchheim, Germany). Acquisition and reconstruction parameters: tube current 150–200 mA, tube voltage of 100 or 120 kV; pitch 0.8; matrix size 512 × 512. Section thickness was set at 5 mm.

**Fig. 1:**
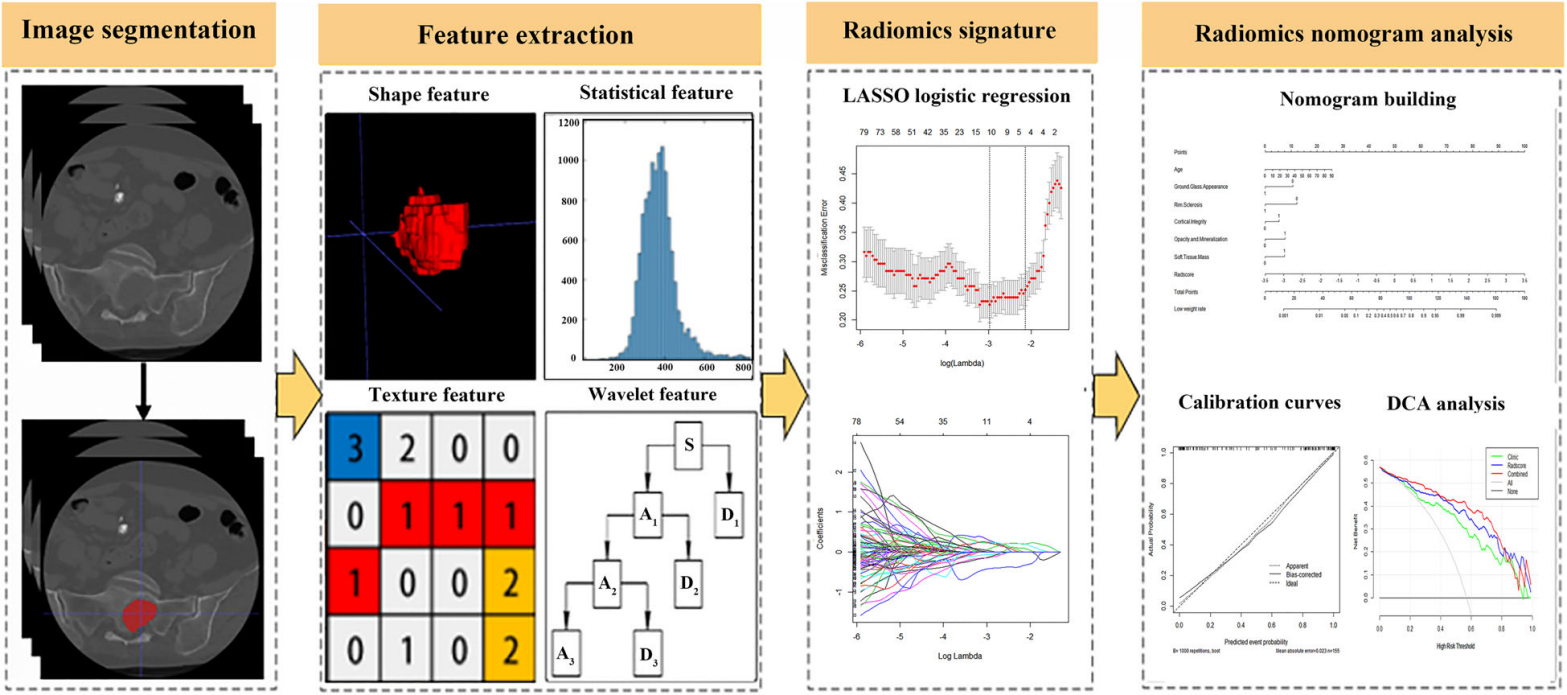
Workflow of the radiomics analysis

The radiomics workflow is shown in Fig. 1. The tumour was evaluated in three dimensions and the segmentation of the tumour regions of interest (ROIs) was based on ITK-SNAP (v.3.8.0 http://www.itksnap.org) open-source software [12]. The ROI was manually segmented layer by layer along the whole tumour region (excluding peritumoral oedema), and only the largest of the multiple lesions was sketched. This step was processed by a radiologist with 7 years of experience; the ROIs were verified a week later by another radiologist with 14 years of experience. Any difference was re-delineated after consultation. The intraobserver reproducibility was reflected according to the intra-class correlation coefficients (ICCs) and we chose 40 random ROI segmentations for calculating ICCs.

### Image normalisation and feature extraction

At the beginning of extraction, pre-processing was necessary to improve discrimination between texture features. In the first step, data normalisation and grey-level discretisation were conducted to enhance the discrimination of different sets and improve the convergence rate of the model. Then, an eight-level quantisation representation was used to resample the acquisition area to a specific isotropic resolution (voxel size = 1 × 1 × 1 mm^3^) at a consistent angle to the plane resolution [13].

Feature extraction was processed on the SlicerRadiomics model in the 3D Slicer Radiomics Extension Pack (v.4.10.2 https://www.slicer.org/). High-pass or low-pass wavelet filters, LoG filters with different λ-parameters (λ = 0.5, 1.0, 1.5) and wavelet-based processes were used for pre-processing the original CT images. We set the fixed bin width at 25 HU to discretise the voxel intensity values. We then extracted the original CT image and the radiomics characteristics of segmented lesions, including the first-order characteristics based on CT value or pre-processed image pixel values, the grey level co-occurrence matrix, grey-level run-length matrix, grey-level size zone matrix and neighbourhood grey-tone difference matrix, which were described the morphological characteristics of tumour form and the internal and surface texture feature. There were 1130 CT features drawn from lesions, and we used Ζ- scores to standardise into for a gaussian distribution. Additionally, the ComBat method technique was used to eliminate differences in image features caused by variations in the parameters of different CT devices [14]. Finally, we calculated ICCs of the extracted features, which were based on CT images; any feature with an ICC > 0.75 was included in the subsequent analysis.

### CT morphologic characteristics

CT data were reconstructed using bone algorithms (window width 1500–2500 HU, window level 280–400 HU) and evaluated in axial orientation. Two radiologists with 7 and 14 years of experience in skeletal muscle CT diagnosis independently viewed and recorded all CT images. If they had different opinions, they reached a consensus through consultation. The CT features they recorded were 1) location (the location of the main tumour lesion within the medullary cavity or cortex), 2) number (number of tumour lesions, solitary or multiple), 3) margins (well- or ill-defined), 4) expansion (ratio of length to diameter, < 1 or > 1), 5) ground-glass appearance, 6) rim sclerosis, 7) cortical integrity, 8) residual bony ridge, 9) periosteal reaction, 10) cortical destruction, 11) soft tissue mass and 12) adjacent tissue involvement. These CT features were selected for analysis based on previous studies [15, 16].

### Development of the radiomics signature, clinical model, and radiomics nomogram

Subsequent analysis was executed on R software (3.3.1 version, http://www.R-project.org). Dimensional reduction of the dataset was conducted using the least absolute shrinkage and selection operator (LASSO) regression model. The radiomics signature was formed by the linear combination of the features selected by LASSO regression and the product of the corresponding weighting coefficient [17], while the radiomics score (rad-score) was also calculated. The receiver operating characteristic curve was used to evaluate the performance of the model in distinguishing bone tumours. *P* < 0.05 was considered statistically significant. The area under the receiver operator characteristic curve (AUC) was used to predict the accuracy of the radiomics signatures of both the training sets and validation sets. The formula used to calculate rad-score was:

1 $$ Rad- score=-0.1323\ast Original\_ shape\_ Sphericity+0.2145\ast Original\_ shape\_ Maximum\ 2D\  Diameter\ Slice+0.068\ast Log\left( sigma-1-5- mm\right)\_ glcm\_ Maximum\ Probability+0.304\ast Log\left( sigma-1-5- mm\right)\_ glcm\_ Joint\ Energy+0.2414\ast Log\left( sigma-1-5- mm\right)\_ glcm\_ Idn-0.4306\ast Log\left( sigma-1-5- mm\right)\_ first\ order\ Robust\ Mean\ Absolute\ Deviation+0.2387\ast Log\left( sigma-1-5- mm\right)\_ first order\_10 Percentile+0.0317\ast Wavelet\_ LHH\_ glszm\_ Small\ Area\ Emphasis-0.1469\ast Wavelet\_ HLH\_ gldm\_ Dependence\ Variance-0.0667\ast Wavelet\_ LLL\_ first\ order\_ Mean\ Absolute\ Deviation+0.3233. $$

The clinical risk factors for assessing bone tumours were analysed by univariate logistic regression. The factors for which *P* < 0.05 in multivariate logistic regression were used in the clinical model. In logistic regression, the Akaike information criterion was used as the stop indication of the stepwise method. Next, the collinearity was evaluated using the variance inflation factor; the condition of variance inflation factor > 10 was used as an exclusion criterion. The radiomics nomogram was built on the basis of the aforementioned clinical parameters.

### Performance and validation of the radiomics nomogram

A calibration curve was used to evaluate the calibration of the nomogram; the Hosmer–Lemeshow test was used to assess the goodness-of-fit of the nomogram. Data from the validation set was used to verify the validation of nomogram and calculate the rad-score. Then, the AUC was measured using the calibration curve and the Hosmer–Lemeshow test to assess the effectiveness of the radiomics nomogram model. Finally, the Delong test was used to compare AUCs between sets; *P* < 0.05 was regarded as statistically significant.

Decision curve analysis (DCA) is an approach for evaluating the availability and efficiency of radiomics models, with the ability to graphically display the “net benefit” of the radiomics model [18]. Based on regression prediction analysis, a loss function was introduced into the DCA to calculate the threshold probability of the validation set. Furthermore, the net reclassification improvement (NRI) and total integrated discrimination index (IDI) were used to compare prediction performance between groups [19]. The value of NRI can be positive or negative. A positive value indicates that the model provides a net improvement in clinical decision-making for patients with bone tumours.

### Statistical analysis

Using R software to perform statistical analyses, the Kolmogorov–Smirnov test was first conducted to examine whether these texture feature parameters followed a normal distribution. For continuous variables, we used an independent-samples *t*-test and univariate analysis to evaluate whether the feature average values were significantly different between clinical or morphological characteristics and malignancy. The Mann–Whitney *U*-test was applied to examine those non-normally distributed features, while the inter-group categorical variables were compared using Fisher’s exact test or the chi-squared test. *P* < 0.05 was regarded as statistically significant.

We used the “glmnet” package for the analysis of LASSO logistic regression, which was applied to the radiomics features. Each patient’s rad-score was a sum of the product of the final retained features based on the radiomics features with their corresponding coefficients.

Finally, the “rms” package was used to generate nomogram and calibration curves. The AUC represents the optimal cutoff threshold value that was computed; models with larger AUCs had higher prediction efficacy. The “generalhoslem” and “dca. R” packages were used to calculate the Hosmer–Lemeshow test and DCA, respectively.

## Results

### Clinical characteristics

Relevant demographics and CT features were obtained using univariable analysis, as shown in Table 2. The results showed that there were statistically significant differences between the two sets in age and nine CT morphological features (*P* < 0.05).

**Table 2:** Demographic data and CT morphological features

Feature	Benign	Malignant	*P* value
No. of patients	88	118	
Gender			0.087
Male	56	61	
Female	32	57	
Age (mean ± SD)	47.77 ± 19.88	30.30 ± 18.93	< 0.001
< 50 years	69	53	
≥ 50 years	19	65	
Site			0.378
Head and neck	15	13	
Upper extremity	14	20	
Trunk wall	1	9	
Spine	8	42	
Lower extremity	50	34	
Location			0.601
Within the medullary cavity	49	70	
Within the cortex	39	48	
Number			0.422
Solitary	75	105	
Multiple	13	13	
Expansion			0.012
< 1	75	83	
> 1	13	35	
Margin			< 0.001
Well-defined	71	49	
Ill-defined	17	69	
Ground glass appearance			0.038
-	68	104	
+	20	14	
Rim sclerosis			< 0.001
-	43	98	
+	45	20	
Cortical integrity			< 0.001
-	63	33	
+	25	85	
Residual bony ridge			0.001
-	65	61	
+	23	57	
Periosteal reaction			0.036
-	79	93	
+	9	25	
Cortical destruction			
-	78	103	0.769
+	10	15	
Soft tissue mass			< 0.001
-	74	62	
+	14	56	
Adjacent tissues involvement			0.041
-	74	85	
+	14	33	

### Clinical modelling

The clinical features found to be significantly different in the benign and malignant bone tumours by univariate analysis are presented in Table 3. These features were selected by multivariate logistic regression to establish the clinical model. The results are listed in Table 4. There were statistically significant differences in age, ground-glass appearance, rim sclerosis, cortical integrity, residual bony ridge and presence or absence of a soft tissue mass between the two groups (*P* < 0.05 each). These six features combined as the final clinical model with an AUC of 0.858 (95% confidence interval [CI] 0.799–0.917) in the training sets and 0.815 (95% CI 0.696–0.934) in the validation sets.

**Table 3:** Positive results of univariate analysis

	Log OR	SE	OR	*P* value
Gender	0.43	0.331	1.53	0.196
Age	0.04	0.009	1.04	< 0.001
Expansion	0.89	0.414	2.44	0.031
Margin	1.70	0.371	5.47	< 0.001
Ground Glass Appearance	−1.01	0.438	0.36	0.021
Rim Sclerosis	−1.66	0.375	0.19	< 0.001
Cortical Integrity	1.66	0.353	5.28	< 0.001
Opacity and Mineralization	1.21	0.357	3.34	0.001
Periosteal reaction	1.00	0.500	2.71	0.046
Soft tissue mass	1.66	0.404	5.23	< 0.001
Adjacent tissues involvement	0.67	0.406	1.95	0.099

**Table 4:** Positive results of multivariate logistic regression analysis

	Log OR	SE	OR	*P* value
Age	0.03	0.011	1.03	0.006
Ground Glass Appearance	− 1.48	0.548	0.23	0.007
Rim Sclerosis	−1.39	0.455	0.25	0.002
Cortical Integrity	1.01	0.429	2.75	0.018
Residual bony ridge	0.99	0.455	2.70	0.029
Soft tissue mass	1.29	0.503	3.63	0.010

*Log OR* Logarithm of Odds Ratio, *SE* Standard deviation, *OR* Odds ratio

### Radiomic feature selection and its performance

**Fig. 2:**
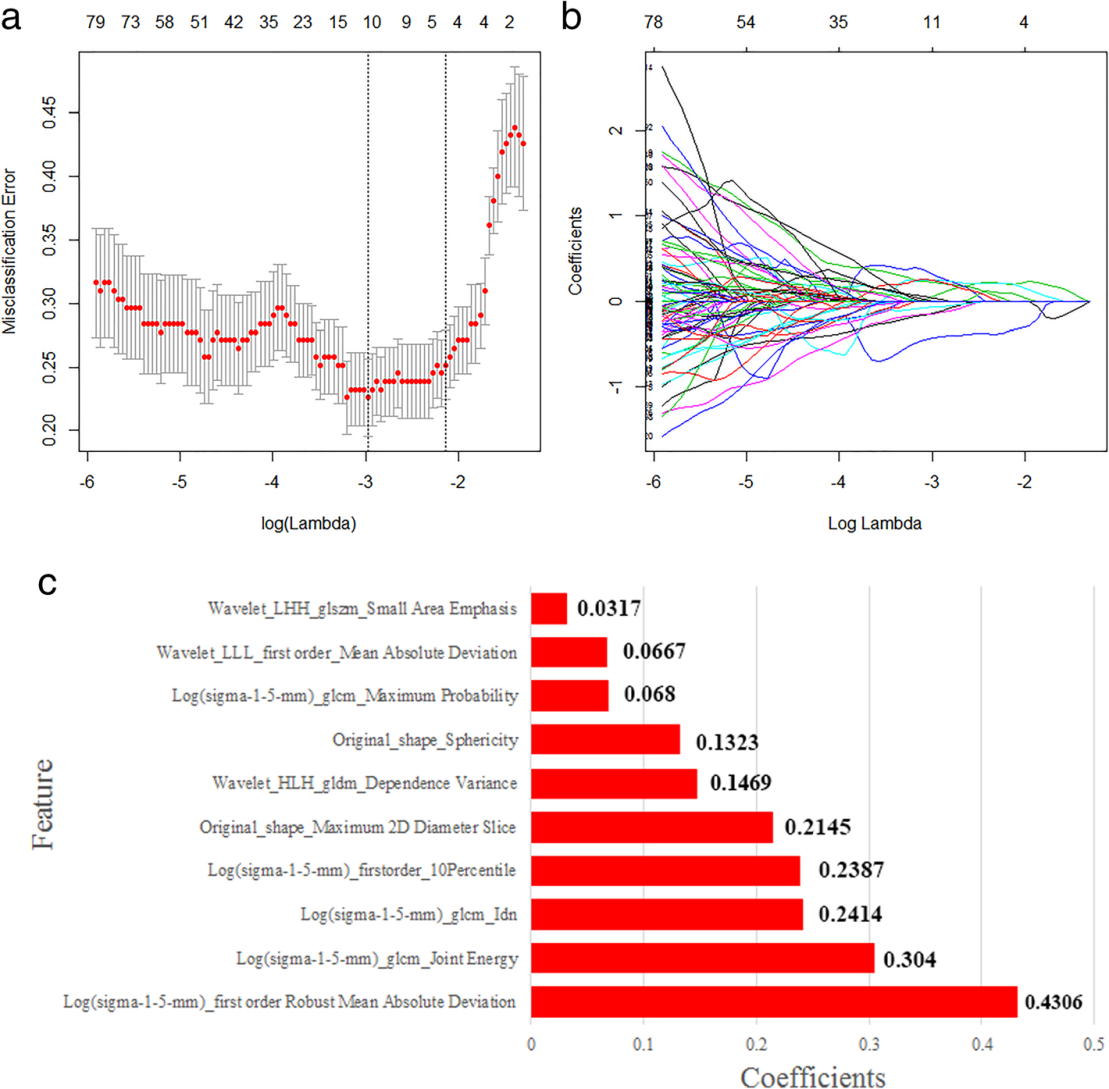
Texture features selected by the LASSO regression model. **a** Tuning parameter (λ) selection in the LASSO model. The top value represents the corresponding characteristic number. In this study, the optimal λ value corresponding to the perpendicular line was selected to obtain 10 features with non-zero coefficients. **b** Variation of LASSO coefficients for different features as modulation parameter (λ value) changes. **c** Contributions of the 10 selected features with nonzero coefficients to the radiomics signature, with their respective coefficient values

In the training set, 10 features were selected by comparing the ICCs calculated through LASSO logistic regression. The process is shown in Fig. 2a and b. Figure 2c shows the features that were filtered. The radiomics scores were calculated to identify benign versus malignant status of bone tumours. The radiomics features showed good predictive accuracy: 0.832 with an AUC of 0.892 (95% CI, 0.842–0.942) in the training set and 0.804 with an AUC of 0.781 (95% CI, 0.643–0.918) in the validation set.

### Validation of the nomogram

**Fig. 3:**
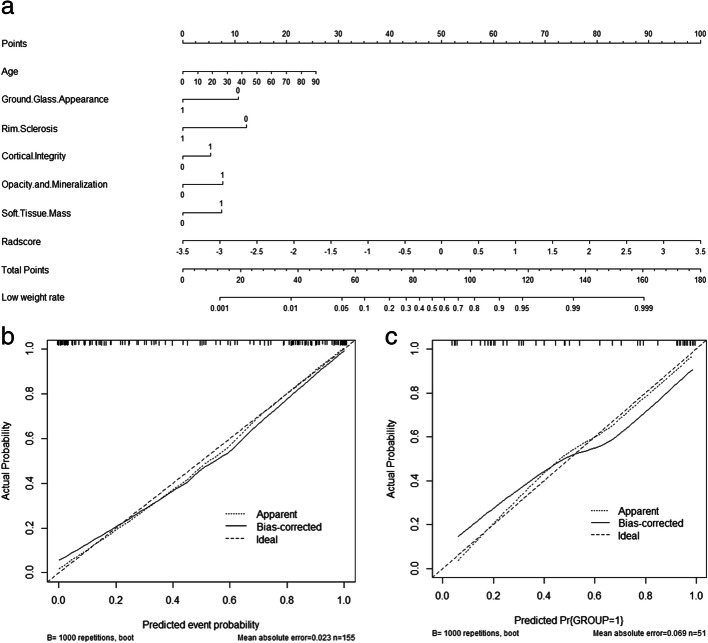
The radiomics nomogram incorporated seven factors of rad-score and clinical features (**a**). Calibration curves of the radiomics nomogram in the training set (**b**) and validation set (**c**). The dotted line indicates the optimal prediction and the solid line represents the real predictive ability of the model. When the solid line gets closer to the dotted line, the nomogram has better performance

A nomogram model was constructed that incorporated the radiomics signature and clinical features derived from previous LASSO logistic regression (Fig. 3a). The performance of the nomogram is shown in Table 5. The calibration curves are shown in Fig. 3b and c; these include the clinical model, radiomics signature, and nomogram. The calibration curve showed good calibration in the training set (Fig. 3b) with a nonsignificant Hosmer–Lemeshow test result (*P* = 0.510), verified by the validation set (Fig. 3c) with *P* = 0.653.

**Table 5:** Results of radiomics nomogram, radiomics signature, and the clinical model predictive ability for distinguishing between malignant and benign bone tumour

Variables	Group	AUC (95% CI)	Accuracy	Sensitivity	Specificity	PPV	NPV
Clinical model	Train	0.858 (0.799–0.917)	0.813	0.876	0.727	0.780	0.814
	Validation	0.815 (0.696–0.934)	0.745	0.586	0.955	0.944	0.636
Radiomics signature	Train	0.892 (0.842–0.942)	0.832	0.854	0.803	0.854	0.803
	Validation	0.781 (0.643–0.918)	0.804	0.793	0.818	0.852	0.750
Radiomics nomogram	Train	0.917 (0.871–0.963)	0.871	0.854	0.894	0.916	0.819
	Validation	0.823 (0.686–0.959)	0.863	0.931	0.773	0.844	0.895

*AUC* Area under the curve, *CI* Confidence interval, *PPV* Positive predictive value, *NPV* Negative predictive value

**Fig. 4:**
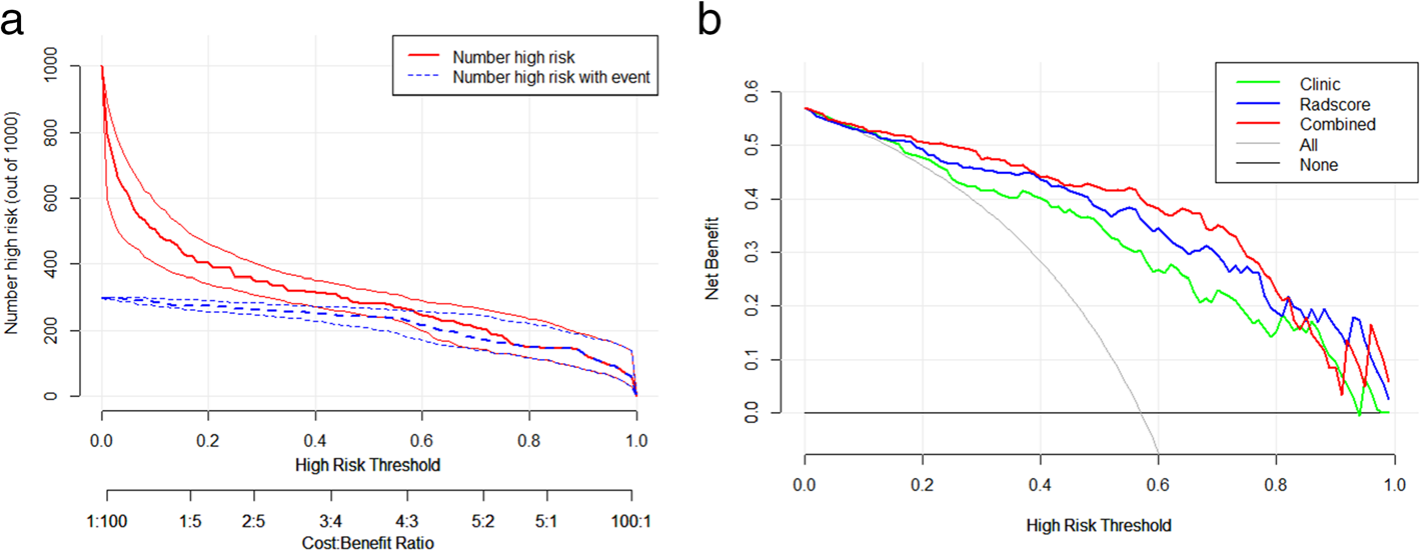
The clinical impact curve and the DCA. **a** In the clinical impact curve, the red solid line indicates the number of patients at high risk with relevant risk threshold, and the blue dotted line indicates that patients with bone tumours that are truly positive for malignancy. This curve showed that the model had a better predictive ability for high-risk bone tumour patients with a range of threshold probability. **b** DCA for clinical model (green line), radiomics signature (blue line), and nomogram (rad line). The grey line is made with the assumption that all tumours are malignant. The black line is made with the assumption that no tumours are malignant. The curve indicates that the net benefit of the nomogram is better than the other cases when the threshold is within the range 0.1–0.8

The results indicated that the radiomics nomogram had better diagnostic performance than the clinical model (AUC: 0.917 vs. 0.858, *P* = 0.008), but the radiomics nomogram diagnostic performance did not differ significantly from that of the radiomics signature model (AUC: 0.917 vs 0.892, *P* = 0.102). DCA and NRI results indicated that the radiomics nomogram model had higher net benefits for clinical decision making, with an NRI of 0.238 (95% CI: 0.07–0.405) and an IDI of 0.163 (95% CI: 0.105–0.222) when comparing between the radiomics nomogram and clinical model, whereas there was an NRI of 0.196 (95% CI: 0.068–0.324) and an IDI of 0.09 (95% CI: 0.046–0.133) when comparing between the radiomics nomogram and radiomics signature (Fig. 4a, b).

## Discussion

It is extremely important and challenging for clinical management and strategy to distinguish benign from malignant bone tumours. We proposed a CT-based nomogram that could distinguish between benign and malignant bone tumours. The nomogram, combined with the radiomics signature, age, ground ground-glass appearance, rim sclerosis, cortical integrity, residual bony ridge, and soft tissue mass, was successful in distinguishing between benign and malignant bone tumours. Our analysis indicated that the radiomics nomogram achieved a relatively good performance compared with the clinical model and the radiomics signature. Moreover, our model could serve as a noninvasive and preoperative method for accurate diagnosis; it could therefore be used to plan therapy for patients with bone tumours.

Morphological features in CT are often used to distinguish between benign and malignant bone tumours. Strobel et al. predicted malignancy in bone based on a combination of ill-defined margins, cortical destruction, and periosteal reactions on CT with accuracy, sensitivity and specificity of 78, 85, and 65%, respectively [16]. Our study achieved greater specificity (74.5, 58.6, 95.5%) at the cost of lower sensitivity. The combination of age, ground ground-glass appearance, rim sclerosis, cortical integrity, residual bony ridge, and presence of a soft tissue mass was proved to be a significant predictor of malignancy in the clinical model in our study. A possible explanation is that rim sclerosis (a predictor of benignity) was probably present because benign bone tumours have a slow growing process, which means that the bone repair process is faster than the destruction process. The presence of a soft-tissue mass was likely to indicate a malignant growth pattern of tumour lesions destroying or permeating through the Haversian canals to violate the surrounding tissue, which was consistently proven in previous studies [20–22]. However, the value of clinical models largely relies on the experience and capabilities of radiologists to precisely interpret CT imaging, which explains why the diagnostic efficiency of the clinical model was lower than that of the radiomics nomogram.

MRI features have also been used to distinguish between malignant and benign bone tumours. The findings by Yu et al. of masses suggestive of malignancy were only 75.8% accurate [23]. Another study by Xu et al. reported an accuracy of 89.8% using diffusion-weighted imaging sequences in distinguishing between benign and malignant orbital masses [24]. Cao. et al. used dynamic contrast-enhanced MRI to distinguish between malignant and benign bone tumours with an accuracy of 90.6% [25]. Masaki et al. used diffusion kurtosis imaging and reported a specificity and sensitivity of 96.3 and 93.8% [26], respectively. These studies showed similar levels of prediction performance as our radiomics nomogram. However, compared with CT scanning, MRI is expensive for equipment, has longer image acquisition times, is easily affected by patient motion, and is contraindicated for patients with metal in their bodies. Thus, we developed a radiomics nomogram based on plain CT images.

Radiomics is derived from imaging but is elevated above imaging. It has rapidly become an approach to transform images into multi-dimensional quantitative data to support clinical decision making [27]. Importantly, radiomics is used in the assessment of tumour characteristics and may replace tissue biopsy in some cases to reflect a more precisive situation of the internal characteristics of tumours [28]. To improve the accuracy of distinguishing benign from malignant bone tumours, we established a radiomics nomogram. This is a graphical calculation tool that can establish a scoring standard on the basis of the regression coefficients of extracted features, thereby accurately predicting the risks of various outcomes. A previous study reported that texture parameters on CT had an accuracy of 77.8% for predicting malignancy in bone lesions [11]. In our study, 10 features were selected by LASSO logistic regression to construct the radiomics signature model. In some studies, LASSO regression has been applied to avoid over-fitting in model construction; accordingly, it is widely used in dimensionality reduction involving high-dimensional data [29]. Therefore, we used LASSO regression to improve the accuracy of the final model. Moreover, Lisson et al. reported that various texture features, such as kurtosis, entropy, and skewness, could distinguish low-grade chondrosarcoma from enchondroma; those findings indicated that texture features could be used for the differentiation of bone tumours [30]. Similarly, on the basis of the coefficient values of each feature obtained in this study, the first-order characteristics (mean absolute deviation, 10th percentile), shape characteristics and grey level co-occurrence matrix characteristics provide greater contributions to our nomogram model. Additionally, we combined our radiomics signature model with a clinical model that included some characteristics that could better reflect the biological characteristics of the tumour (e.g., ground ground-glass appearance, rim sclerosis, cortical integrity, and residual bony ridge). Thus, we established a more accurate nomogram which demonstrated the good applicability prospect of radiomics nomograms in bone tumours. Furthermore, some scholars have recently implemented texture analysis combined with diffusion-weighted imaging and contrast-enhanced T1-weighted MRI in the differentiation of bone tumours or soft tissue tumours; the results have been relatively good [31, 32]. This provides insights for future research, whereby emerging imaging technology can be combined with radiomics (e.g., dynamic contrast-enhanced MRI, intravoxel incoherent motion, and diffusion kurtosis imaging to explore their potential applications in assessment of the skeletal muscle system.

We used ComBat to remove the different CT protocol effects and applied DCA to verify the feasibility of the clinical model, standardising the differences in image features caused by variations in the parameters of different CT devices using the ComBat method. As we know, different scan settings of tube voltage, tube current, thickness, pitch, and matrix may affect the feature values [14]. This method is expected to solve the protocol effect caused by multicentre data and to enhance the reliability of the article. In practice, all imaging acquisition differences from different centres or protocols should be identified and transformed into a uniform standard using ComBat. We used DCA to estimate whether the model had good clinical value in our study. When the radiomics model got a higher “net benefit” in the DCA, a more customised therapy strategy could be employed to improve patients’ prognosis.

Our study had some limitations. First, potential selection bias was inevitable because of its retrospective nature. Second, manual tumour segmentation inevitably encounters irregularities. Therefore, all images were standardised by normalisation to control the variables [33]. Third, we conducted a retrospective study based on single-centre CT imaging of different protocols, so we used the ComBat method to eliminate the protocols’ effect to enable further studies with independent multicentre validation of radiomic models. Finally, our study was a single-centre study with a limited sample of patients with bone tumours; therefore, a multicentre study is necessary to verify the accuracy and efficacy of our nomogram model.

## Conclusions

In conclusion, we developed a radiomics nomogram integrating the clinical model and radiomic features that contribute to the prediction of distinction between benign and malignant bone tumours and supplements the routine clinical strategy.

## Data Availability

The datasets used and/or analysed during the current study are available from the corresponding author on reasonable request.

## Abbreviations

CT: Computed tomography
LASSO: Least absolute shrinkage and selection operator
DCA: Decision curve analysis
IDI: Integrated discrimination index
NRI: Net reclassification improvement
MRI: Magnetic resonance imaging
ROI: Region of interest
ICC: Intra-class correlation coefficient
AUC: Area under the receiver operator characteristic curve
DKI: Diffusion kurtosis imaging
